# Neurological Disease Modeling Using Pluripotent and Multipotent Stem Cells: A Key Step towards Understanding and Treating Mucopolysaccharidoses

**DOI:** 10.3390/biomedicines11041234

**Published:** 2023-04-21

**Authors:** Sofia Carvalho, Juliana Inês Santos, Luciana Moreira, Mariana Gonçalves, Hugo David, Liliana Matos, Marisa Encarnação, Sandra Alves, Maria Francisca Coutinho

**Affiliations:** 1 Research and Development Unit, Department of Human Genetics, National Institute of Health Doutor Ricardo Jorge, INSA I.P., Rua Alexandre Herculano, 321, 4000-055 Porto, Portugal; sofia.carvalho@insa.min-saude.pt (S.C.); juliana.santos@insa.min-saude.pt (J.I.S.); luciana.moreira@insa.min-saude.pt (L.M.); mariana.goncalves@insa.min-saude.pt (M.G.); hugo.david@insa.min-saude.pt (H.D.); liliana.matos@insa.min-saude.pt (L.M.); marisa.encarnacao@insa.min-saude.pt (M.E.); sandra.alves@insa.min-saude.pt (S.A.); 2 Center for the Study of Animal Science-Instituto de Ciências, Tecnologias e Agroambiente da Universidade do Porto, CECA-ICETA, University of Porto, Praça Gomes Teixeira, Apartado 55142, 4051-401 Porto, Portugal; 3 Associate Laboratory for Animal and Veterinary Sciences, AL4AnimalS, Faculdade de Medicina Veterinária Avenida da Universidade Técnica, 1300-477 Lisboa, Portugal; 4 Faculty of Pharmacy, University of Coimbra, Polo das Ciências da Saúde, Azinhaga de SantaComba, 3000-548 Coimbra, Portugal; 5 Biology Department, Faculty of Sciences, University of Porto, Rua do Campo Alegre, 4169-007 Porto, Portugal; 6 Centre for the Research and Technology of Agro-Environmental and Biological Sciences, CITAB, Inov4Agro, University of Trás-os-Montes and Alto Douro, 5000-801 Vila Real, Portugal

**Keywords:** mucopolysaccharidoses, disease modeling, in vitro models, induced pluripotent stem cells (iPSCs), dental pulp stem cells (DPSCs)

## Abstract

Despite extensive research, the links between the accumulation of glycosaminoglycans (GAGs) and the clinical features seen in patients suffering from various forms of mucopolysaccharidoses (MPSs) have yet to be further elucidated. This is particularly true for the neuropathology of these disorders; the neurological symptoms are currently incurable, even in the cases where a disease-specific therapeutic approach does exist. One of the best ways to get insights on the molecular mechanisms driving that pathogenesis is the analysis of patient-derived cells. Yet, not every patient-derived cell recapitulates relevant disease features. For the neuronopathic forms of MPSs, for example, this is particularly evident because of the obvious inability to access live neurons. This scenario changed significantly with the advent of induced pluripotent stem cell (iPSC) technologies. From then on, a series of differentiation protocols to generate neurons from iPSC was developed and extensively used for disease modeling. Currently, human iPSC and iPSC-derived cell models have been generated for several MPSs and numerous lessons were learnt from their analysis. Here we review most of those studies, not only listing the currently available MPS iPSC lines and their derived models, but also summarizing how they were generated and the major information different groups have gathered from their analyses. Finally, and taking into account that iPSC generation is a laborious/expensive protocol that holds significant limitations, we also hypothesize on a tempting alternative to establish MPS patient-derived neuronal cells in a much more expedite way, by taking advantage of the existence of a population of multipotent stem cells in human dental pulp to establish mixed neuronal and glial cultures.

## 1. Introduction

Lysosomal storage disorders (LSDs) are a group of rare diseases caused by mutations in genes that encode lysosomal enzymes, lysosomal membrane proteins, or transporters, and in a few cases caused by other cell proteins that are important for lysosomal function. This triggers an accumulation of undegraded substrates, which ultimately results in a broad range of clinical symptoms. Overall, those symptoms may be highly debilitating and affect multiple organs/systems, including the central nervous system (CNS) [1]. Among the LSDs, which may present with severe neurological phenotypes are mucopo-lysaccharidoses (MPSs), which are caused by impaired degradation of glycosaminoglycans (GAGs), with consequent intralysosomal accumulation of undegraded products [2]. Quite remarkably, none of the available therapies for this sub-group of disorders are effective against the neurological symptoms. Instead, they are limited to treating non-neurological symptoms [3]. Thus, there is an urgent need for the development of new ones that can tackle the neuronal pathogenesis. A crucial step towards the development of those approaches is the existence of suitable disease models, which can be used to further understand the pathophysiological mechanisms that underlie the phenotype and to adequately test those therapeutic strategies in vitro. Here, we review some of those models and the major results that other groups have published on the pathophysiological mechanisms underlying this particular subset of LSDs. To start with, we will highlight the different patient samples they used and the protocols they relied on. Particular attention will be given to the potential of induced pluripotent stem cells (iPSCs) to mimic disease-relevant phenotypes, and to the methods others have used to assess them. Finally, we will also mention a few studies, which have provided in vitro proof of principle on the potential of ex vivo genetically corrected iPSC-derived cells for therapeutic purposes.

Overall, the results reviewed here strongly support the utility of iPSCs for the study of MPSs. Still, iPSC generation is a laborious and expensive protocol. Furthermore, the use of iPSCs has a number of limitations, which should not be ignored. That is why in our lab we are addressing the question of whether alternative sources of stem cells (SCs) may exist, holding a similar potential for disease modeling in these rare yet life-threatening genetic disorders. In fact, recent studies have shown that dental pulp provides a niche for diverse arrays of mesenchymal stem cells (MSCs), and they are now being established in our laboratory for the study of LSDs, particularly MPSs. This approach is non-invasive, cost-effective, and can be established in any laboratory with standard cell culture conditions. Additionally, as we will briefly highlight in this manuscript, it may provide another potentially effective approach for investigating cellular and gene expression changes that occur in these monogenic diseases.

## 2. Mucopolysaccharidoses

Among the LSDs in need for better and more effective therapeutic options are the mucopolysaccharidoses (MPSs). The MPS subgroup includes seven different disease types, all of them accumulating glycosaminoglycans (GAGs) as the primary substrate. A brief overview of each individual disorder is given below. 

MPS I is one of the most common forms of MPS and the first MPS type treated with enzyme replacement therapy (ERT; available since 2003) [4]. At a clinical level, MPS I may be divided into three subtypes: Hurler (OMIM #607014), Hurler–Scheie (OMIM #607015), and Scheie (OMIM #607016) depending on the disease severity [5]. Regardless of the clinical presentation, *IDUA* is the affected gene in this disorder. Mutations in this gene, which encodes for α-L-iduronidase (IDUA; EC 3.2.1.76), lead to an enzyme deficiency that ultimately results in heparan and dermatan sulfate (HS and DS, respectively) accumulation [6]. Currently, there are two possible therapeutic options for MPS I, ERT and hematopoietic stem cell transplantation (HSCT), which is only used in the most severe form of the disease and, preferably in the first years of life [7]. Regarding ERT, there is only one recombinant enzyme approved for MPS I: laronidase (Aldurazyme^®^, Genzyme). As every other ERT, this recombinant enzyme is injected into the blood circulation, which leads to the correction of the enzyme deficiency in various organs and tissues, except for the brain, since it does not cross the blood brain barrier (BBB) [8,9]. 

MPS II (OMIM #309900), or Hunter syndrome, is the only X-linked MPS disease; all the other MPSs are autosomal. Two forms of the disease may be distinguished, neuronopathic and non-neuronopathic, whereby the most severe forms are CNS-associated [10]. Both forms are caused by mutations in the *IDS* gene, which encodes the enzyme iduronate 2-sulfatase (IDS; EC 3.1.6.13), leading to the accumulation of two substrates: HS and DS. Regarding MPS II therapeutics, ERT with idursulfase (Elaprase^®^, Takeda Pharmaceutical Company) is the first choice for patients with this condition [11]. 

MPS type III, also known as Sanfilippo syndrome, may be subdivided into four subtypes: IIIA (OMIM #252900), IIIB (OMIM #252920), IIIC (OMIM #252930), and IIID (OMIM #252940). Each particular subtype is associated with a unique enzymatic defect. MPS IIIA is caused by the deficiency of the enzyme heparan-N-sulfatase (SGSH, EC 3.10.1.1); MPS IIIB, in turn, is caused by defects in the enzyme N-acetylglucosaminidase (NAGLU, EC 3.2.1.50); in MPS IIIC the protein involved is the transmembrane enzyme, acetyl-CoA:Glucosamine N-acetyltransferase (HGSNAT, EC 2.3.1.78); and, finally, MPS IIID is caused by defects in N-acetyl-glucosamine-6-sulfatase (GNS, EC 3.1.6.14). All MPS III subtypes are associated with a severe deterioration of neurological function [12], which results in a number of clinical symptoms either directly or indirectly related to a CNS dysfunction [13,14]. Unfortunately, there is no approved treatment for these neurological diseases. On the one hand, while it has already been attempted by several different teams, HSCT has proven virtually no benefit over the neurocognitive symptoms [15,16,17,18,19]. On the other hand, ERT is hard to apply, since classically formulated enzymes do not penetrate the CNS. Moreover, in the case of MPS IIIC, for example, ERT is not an option, since the deficient enzyme is a transmembrane protein. There are, however, teams attempting brain-specific delivery of both ERT and chemical compounds for MPS type III. In general, there are three strategies to increase the delivery (reviewed in [20]): enzymatic modulation, route(s) of administration [21,22,23], and increase in enzyme dosage. In addition, cellular and genetic therapies represent approaches that have gained importance when it comes to BBB delivery (reviewed in [24]). 

There are two different forms of MPS IV, each one caused by a single enzymatic defect: N-acetyl-galactosamine-6-sulfatase (GALNS; EC 3.1.6.4) deficiency underlies MPS IVA (OMIM #253000) while β-galactosidase (EC 3.2.1.23) defects cause MPS IVB (OMIM #253010). The involved genes are *GALNS* and *GLB1*, respectively. Unlike MPS III, which is almost exclusively a neurological syndrome, the skeleton is the main affected system in MPS IV, with the substrates (keratan sulfate (KS) and chondroitin-6-sulfate (C6S)) accumulating predominantly in the cartilage and bones [25]. The only FDA-approved treatment for MPS IV is elosulfase alfa (Vimizim^®^; BioMarin Pharmaceutical Inc.) which is used to treat MPS IVA patients. All other options are symptomatic and mostly consist of surgical approaches to prevent spinal cord damage or other skeletal issues, for example spinal decompression surgery [26]. 

Yet another form of MPS, usually coined Maroteaux–Lamy Syndrome, is MPS type VI (OMIM #253220), caused by mutations in the *ARSB* gene [27]. Even though being a multisystemic condition, MPS VI does not affect intelligence, and, as in the Morquio syndrome, the skeleton is the most affected system [28]. To counteract the DS storage promoted by the deficiency of arylsulfatase B (EC 3.1.6.12) activity, galsulfase (Naglazyme^®^, BioMarin Pharmaceutical Inc.) is the drug approved for and currently employed in patients. HSCT may also be possible; however, additional safety studies are needed [29,30,31]. 

MPS type VII (OMIM #253220), or Sly syndrome, is an extremely rare form of MPS, which occurs with an estimated frequency of 0.02 [32,33,34,35] to 0.29 [36] per 100,000 live births (reviewed in [37]). Several systems/organs are involved in this disease with clinical features affecting organs as diverse as the eyes, lungs, heart, musculoskeletal system, spleen, etc. There may also be a neurological involvement as testified by recurrent observations of limited vocabulary and mental retardation in several MPS VII patients [38]. Overall, these symptoms are caused by an ubiquitous accumulation of several different GAGs, namely DS, HS, and CS, as a consequence of the deficient activity of β-glucuronidase (GUS: β-D-glucuronoside glucuronosohydrolase, EC 3.2.1.31), which is encoded by the *GUSB* gene [39]. The approved drug for this pathology is vestronidase alfa (Mepsevii™, Ultragenyx Pharmaceutical Inc.), which is indicated in both pediatric and adult cases [40]. 

Finally, MPS IX, or Natowicz disease (OMIM #601492), is an ultra-rare disorder. The first report was published in 1996, with the described patient presenting a number of clinical manifestations associated to joint and skeletal systems [41]. This disorder is caused by a deficiency in the enzyme hyaluronidase 1 (HYAL1; EC 3.2.1.35) due to mutations in the *HYAL1* gene, which leads to the accumulation of yet another substrate: hyaluronan. Due to the rareness of the disorder, very few mutations have been reported to date, and a possible treatment is very challenging [42]. 

In general, even though the molecular bases and biochemical defects underlying MPS diseases are well defined, knowledge is still lacking on the pathophysiological mechanisms that actually trigger the appearance of different symptoms in the different organs and systems. Furthermore, even though over the last few decades much has been learnt from the study of individual patients and, particularly, from the generation and extensive characterization of bona fide in vivo models, truth is, we still do not fully understand the whole physiological cascade, which underlies some of MPSs’ most challenging phenotypes, namely those which affect the CNS. This is particularly relevant since no therapeutic exists to ameliorate them. Still, finding an in vitro model that could recapitulate the disease-relevant features is also challenging since live neurons are inaccessible cells. Indeed, for almost a century, patient-derived fibroblasts were gold standard for in vitro studies of MPSs, as for all other LSDs. These cells are relatively easy to access, since a simple skin biopsy is enough to obtain them and, remarkably, they do display the cellular phenotype that actually coined these diseases as “storage” disorders: the presence of undegraded or partially degraded substrates. Nevertheless, fibroblasts may also fail to recapitulate disease-relevant features, which are only evident in other particular cell types of higher pathological significance, such as neurons. A viable option is to generate the neurons from a patient-derived cell line, which involves extracting cells from the patient and differentiating them into neuronal cells. Indeed, there are two possible ways to perform this process: iPSCs and mesenchymal stem cells (MSCs) from the patient.

## 3. Modeling Mucopolysaccharidoses with Induced Pluripotent Stem Cells (iPSCs)

Human iPSC generation in particular started its journey in 2007, when Yamanaka et al. [43] first generated those cells from human somatic fibroblasts using a remarkable method, which relies on the retroviral transduction of four independent transcription factors into patients’ fibroblasts: Oct-3/4, Sox2, Klf4, and c-Myc. Remarkably, the cells that resulted from this experiment showed numerous similarities with human embryonic stem cells (hESCs), including morphology, proliferative capacity, gene expression patterns, and in vitro differentiation potential. Ever since this hallmark report was published, the search for novel and improved protocols for cell reprogramming advanced at an outstanding pace, with various optimizations being published in order to generate virtually every cell of interest from iPSCs of different origins [44]. 

Over the past few years, in vitro models derived from iPSCs have been unraveling some enigmatic aspects of MPSs. In particular, the subtypes that present neurological involvement appear as the ones with the greatest need for additional knowledge and new therapeutic solutions. 

**Figure 1:**
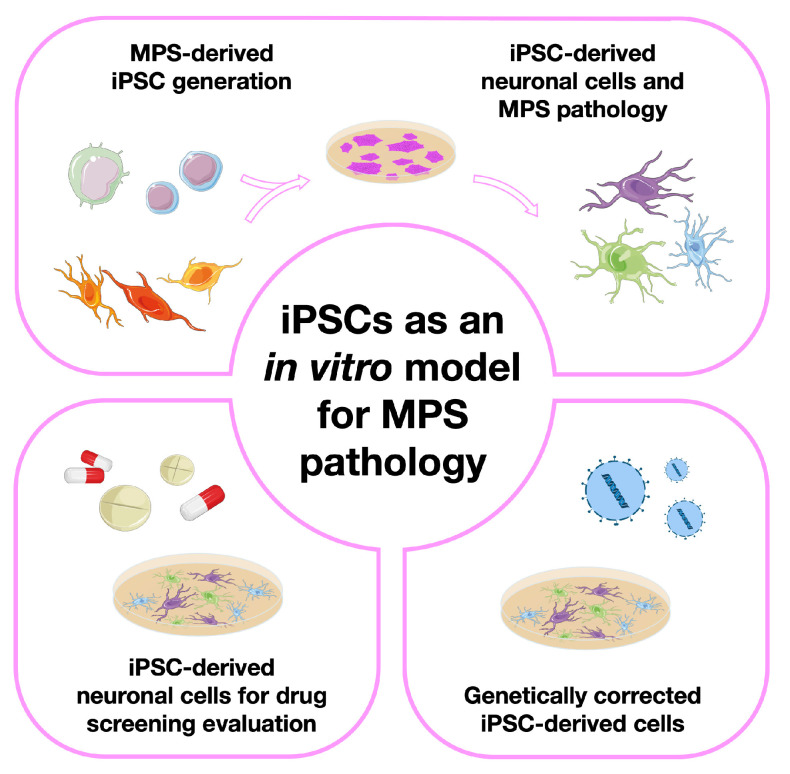
The four major types of studies involving the generation and characterization of induced pluripotent stem cells (iPSCs) as in vitro models for Mucopolysaccharidoses (MPSs), grouped together according to their ultimate aims: (1) papers on iPSC generation and characterization; (2) papers describing their subsequent differentiation into different types of neuronal cells; (3) papers on the use of iPSCs and their derived cell models for drug screening; and (4) papers on the use of genetically corrected iPSCs for therapeutic purposes.

Here, we will review numerous studies attempting not only MPS-derived iPSC generation, but also their subsequent differentiation into relevant cell types. We have divided those studies into four major groups, each one of them having a dedicated section in this review (Figure 1). First, we will address the papers in which only iPSCs were generated, briefly discussing the methods used to characterize them. Then, we will focus on those papers where iPSCs were further differentiated into either neural precursor cells or totally differentiated neurons, highlighting the disease modeling potential of those lines by showing the numerous pathophysiological insights one can get with a few simple cellular assays. Then, we will go through the papers where those cells were used for in vitro drug screening, commenting not only on the results obtained but also on the advantages or disadvantages of the use of those particular cells for therapy development. Finally, in the last iPSC-devoted section, we will refer to a few studies where the therapeutic potential of these particular SCs was addressed. Meaning: we will summarize the papers where, instead of generating iPSCs to further understand one particular disorder or genotype or to serve as a drug-screening platform, the authors have actually created them for gene therapy.

### 3.1. The Basic Studies: iPSC Generation from Different MPS Patient-Derived Cell Sources

The first MPS-derived iPSCs were generated in 2011 when Thomas Lemonnier and colleagues [45] reprogrammed fibroblasts from two patients suffering from MPS IIIB into pluripotent stem cells (PSCs). As in any other iPSC generation report, the resulting SCs were extensively analyzed and characterized. In this particular study, the authors confirmed a positive expression of three particular markers (SSEA4, Nanog, and TRA-1-60) and the differentiation ability of those cells, thus proving their pluripotent nature. Additionally, the authors have also provided information on the karyotype presented by those cells. This is a relevant assessment whenever a novel iPSC line is generated but it should also be considered later on, when using the same iPSC line after several passages, or after having one particular iPSC cell line in culture for a long period. In fact, long-term iPSC culture has already been shown to have a number of drawbacks, as cells tend to accumulate chromosomal abnormalities and changes in gene expression or cellular functions [46,47,48]. Eventually, this increases the risk of tumorigenicity. Altogether, those genomic alterations represent a source of potential risks to many final applications of iPSCs making it is crucial to monitor the genomic integrity of iPSC lines [49]. That is why iPSC karyotype analysis is such an important step in the validation of this type of cell models, and nowadays considered a routine procedure by all the groups working with iPSC technology [45].

However, these were not the only published MPS IIIB-derived iPSCs reported in the literature so far. Two other MPS IIIB patient-derived iPSC lines were generated from skin fibroblasts by Vallejo-Diez et al. in 2018 [50]. In that particular study, additional pluripotency markers besides the previously referred Nanog and TRA-1-60 were also assessed: Oct-3/4, Sox2, and TRA-1-81 were also analyzed. As in the previous study, the karyotype was also assessed, and the associated mutation confirmed. However, the authors actually went one step further in terms of SC characterization, analyzing the differentiation ability of the generated iPSCs. Cells derived from embryoid bodies were differentiated into three germ layers and specific markers were evaluated. The same characterization was carried out for MPS IIIA-derived cells, where the same team was the first to create patient-derived iPSCs for that particular disease [51].

Regarding MPS IIIC, Noelia Benetò et al. also generated iPSC lines, but this time using a slightly different protocol from the previously described ones. Instead of using patient-derived cell lines, these authors created isogenic *HGSNAT*-mutated lines from healthy iPSCs using CRISPR/Cas9. This technology allows to create such lines from human cells, which have the genetic background of the wild-type cells but differ by the genetic modification of interest. These isogenic pairs are powerful tools for understanding gene function. While circumventing confounding effects of the genetic background, they allow for genotype–phenotype correlation studies [52]. To prove the reliability of this model, the authors measured HGSNAT enzyme activity and assessed the differentiation capacity of the generated SCs. This last parameter was studied by inducing the formation of embryoid bodies and their subsequent differentiation into three germ layers. The formation of these structures is a characteristic of pluripotent SCs and serves as a platform for the intended differentiations [53].

Still, even though the neurological involvement is a major hallmark of the Sanfilippo syndrome, almost every other MPS may present with severe neurological symptoms. Type I and II in particular even have specifically recognized clinical forms where the CNS is strongly affected, presenting with major clinical symptoms. Thus, these disorders would also strongly benefit from the development of appropriate neuronal cell models to study them. Furthermore, the models would also allow for tissue- or cell-specific drug screening assays. Generating iPSC lines from those disorders is a rational step towards that first goal, and that is probably one of the reasons why iPSC lines from both disorders have also been created and subsequently published in the past few years. 

Regarding MPS type II, in 2016, Eszter Varga et al. collected peripheral blood mononuclear cells (PBMCs) from 1-, 3-, and 7-year old phenotypically affected patients [54,55,56] and from an unaffected 39-year old woman with a carrier mutation [57]. Then, all patients’ PBMCs were subjected to induction of the pluripotent stage, resulting in disease-specific iPSCs, which were extensively characterized as required for the technology itself. 

Last but not least, MPS type I has also been modeled with the help of this revolutionary technology. In 2019, Lito S. et al. [58] and Suga M et al. [59] reprogrammed and characterized dermal fibroblasts and PBMCs, respectively, into iPSC lines. The fibroblast-derived pluripotent cells were obtained from a patient with the Hurler form of the disease, whereas PBMCs were collected from a patient suffering from Scheie.

### 3.2. Moving One Step Further: Generation of Neuronal Models from MPS-Derived iPSCs

As we already referred to, the neurological involvement, which places such a tremendous burden on patients suffering from several forms of MPS, may be further explored by differentiating iPSCs into different types of neuronal or pre-neuronal populations. In fact, most works published until now are not only focused on reprogramming different types of patient-derived cells (namely fibroblasts and PBMCs) but also on the differentiation step, searching for disease-relevant features in those cells. Ultimately, these models may also allow for the discovery of novel hallmarks related or non-related to neuropathology. Considering the intrinsic nature of all MPSs, lysosomal pathology is probably the most crucial parameter to study, since the enzymatic defect will primarily affect these organelles. 

Thus, when it comes to disease-phenotype assessments, some markers have been particularly relevant in the LSD field, namely the lysosome-associated membrane proteins 1 and 2 (LAMP-1 and LAMP-2). These two proteins are heavily investigated since they represent the major components of the lysosome membrane. For example, in the study involving the first MPS-derived iPSCs [45], which were generated from MPS IIIB patients’ samples, the accumulation of storage lesions was intensively analyzed through LAMP-1 and Golgi matrix protein 130 (GM130) detection. A prominent fluorescence of both markers was detected in patient-derived iPSCs, and the vesicles observed using microscopy were revealed to have a heterogenous content. This was actually the first study to describe Golgi-complex impairment in the MPS pathology. Most importantly, beyond iPSC generation, this group also investigated the differentiation into neural stem cells (NSCs) by adding specific growth factors to the original iPSC culture, namely fibroblast growth factor 2 (FGF2) and endothelial growth factor (EGF). When this protocol was initiated, the development of neurospheres became evident and after 2 weeks of non-adherent growth, the authors measured both the expression of Nestin (a neural progenitor marker) and total GAG storage, observing increased storage levels. Interestingly, the higher LAMP-1 and GM130 expression previously seen in iPSCs did not translate to the floating neurospheres. However, the gene expression profile showed significant alterations in several pathways including the Wnt signal transduction pathway and transforming growth factor β (TGFβ) signaling, as well as genes encoding proteins associated with cell adhesion, Golgi apparatus, and lysosomes. Curiously, higher LAMP-1 and GM130 fluorescence was seen again as soon as neurosphere adhesion was performed, and during the final process of neuronal differentiation. This observation was also accompanied by vesicle storage positive for LAMP-1 and Ganglioside GM3. These results reflect the existence of a modest cellular pathology during the neurodifferentiation of this iPSC model. This study was the first comprehensive characterization of MPS-affected neuronal cells in vitro [45].

To the best of our knowledge, the second report on the differentiation of MPS-derived iPSCs into neuronal cells was the work of Bruyerè and collaborators, in 2015 [60], where these authors correlated two independent models of the disease: one in vitro and another in vivo. For the in vitro studies they used patient- and control-derived iPSCs, further differentiated into neural precursor cells (NPCs), while for the in vivo differences they used a mouse model. Their goal was to investigate how HS saccharide accumulation impacts focal adhesions (FAs). They saw that activation of FAs occurred when neural cells from healthy individuals were submitted to exogenous soluble HS fragments. Consequently, this activation becomes constitutive in MPS IIIB, since those fragments are accumulated. Constitutive activation of FA, in turn, affects the polarization as well as the oriented migration of those cells [60].

Later, in 2015, Canals et al. [61] performed the differentiation of MPS IIIC-derived iPSCs into neuronal cells. Their goal was to verify if early functional alterations could be visible before the appearance of disease-related phenotypes. Briefly, iPSC lines generated spherical neural masses (SNMs), whose expression patterns included PAX6, Nestin, and Sox2. The existence of active neurons was also proven by the presence of microtubule-associated protein 2 (MAP2) and synapsin (SYP), which are dendritic and synaptic markers, respectively. In addition to the formation of mature neurons, an astrocytic-related marker, glial fibrillary acidic protein (GFAP), was noticed. That observation further reinforced the neurogenic capacity of these cells. Regarding the neuronal cultures generated, as expected, GAG accumulation was shown to have a progressive pattern, becoming significant only after 9 weeks. These observations document a marked difference between the patients’ fibroblasts and iPSC-derived neurons: the patients’ fibroblasts presented a double amount of accumulated GAGs, right from the first cell culture, when compared to iPSCs-derived neurons. Network activities were also evaluated to verify whether there were differences between Sanfilippo- and control-iPSC-derived neurons. Through calcium imaging, the spontaneous activity of Sanfilippo-derived neurons was shown to gradually decrease between 6 and 9 weeks. Concerning degradation of effective connectivity, which was determined by identifying causal influences among neurons through generalized transfer entropy, an information theory method that allows drawing a functional map of neuronal interactions in the network, the authors reported that, quite differently from the controls analyzed, in the Sanfilippo neurons strong connections were only established within a subset of neurons, the rest of them remaining disconnected or poorly connected [61]. 

At a technical level, the authors used two different protocols, one relying on neuronal induction medium without any extra supplementation, and another where that medium was supplemented with N2 and B27, two chemically defined supplements recommended for growth and survival of neuronal cells, and observed significant differences in the time it took for them to generate neurons. In fact, while it took several weeks in neuronal induction medium for mature neurons to arise, when supplementing that same medium with N2 and B27, it took only 3–5 weeks to distinguish synapses between neurons. Moreover, the neuronal activity and effective connectivity analyses they performed were nicely designed and described, and could be applicable to virtually any other neurodegenerative disease in which iPSC-based models are available [61].

Five years later, Benetó et al. [62] took advantage of the existence of a few previously reported iPSC lines to generate neuronal and astrocytic models of Sanfilippo syndrome type C for disease modeling and drug development: two isogenic MPS IIIC mutant lines [61], one wild-type control (from a healthy donor), and one MPS IIIC-derived line [53]. Again, all four lines were differentiated into neurons and astrocytes through lentiviral transduction and promoting the overexpression of neurogenin 2 (Ngn2) in the case of neurons (named iNs) and Sox2/Nuclear Factor one B (NfIb) in the case of astrocytes (named iAs). To confirm cell identity, the authors performed a characterization of the specific markers. In the generated neurons, they detected an increase in neural stem cell markers, namely tubulin β-3 (TUBB3), SYP, MAP2, and neuron-specific class III β-tubulin (Tuj1). In the astrocytes, they observed that the expression of astrocytic-specific genes namely GFAP, aldehyde dehydrogenase 1 family member L1 (ALDH1L1), calcium-binding protein B (S100B), and vimentin (VIM) increased during the astrocytic differentiation. In addition, disease-relevant features were assessed through LAMP-2 staining and HS quantification. In the LAMP-2 immunocytochemistry assays, the authors clearly saw an intensity increase in all disease lines compared to the wild-type one. In the case of HS accumulation, they only presented results for neurons, where, as expected, increased substrate storage could be observed [62].

Still in Sanfilippo syndrome, for its most frequent type MPS IIIA, a comprehensive study was carried out by R. J. Lehmann et al. in 2021 [63], to investigate the ability of fibroblasts-derived iPSCs to differentiate into a neuronal cell line and discover intrinsic mechanisms of the disease. After properly characterizing the pluripotent phase, the authors performed a neurodifferentiation protocol. Two main parameters were assessed: the FGF2 signaling pathway and the neurogenesis process. Interestingly, in the beginning of this study a curious fact was noticed: when the FGF2 supplement was added to the medium, the proliferation rate of the MPS IIIA iPSC-derived NPC culture increased significantly. Remarkably, however, even with the supplementary FGF2, the signaling pathway of this factor is still reduced when compared to controls. So, understanding this event became a priority for this team. In fact, the FGF2 signaling pathway only occurs when this factor binds to a possible receptor. Since FGF2 also binds to HS, this may suggest that this GAG plays a key role in neurogenesis and in the homeostasis of the CNS. Taking this into account, the subsequent step was to investigate the relationship between the accumulated HS in MPS IIIA and the lower proliferation rate. They verified that the affinity of HS MPS IIIA to FGF2 was similar to the HS present in the control, meaning that the accumulation does not alter the affinity. So, a possible explanation for decreased FGF2 signaling is that since the FGF2 binds to the accumulated HS, it does not interact with the proper receptors (cell-surface HS and fibroblast growth factor receptors), thus affecting not only cell proliferation without supplementary FGF2 but also the signaling pathway. To investigate the disorder’s impact on the neurogenesis process, control and disease cells were analyzed regarding both morphological parameters and expression patterns. At a structural level, the formation of cell body aggregation and cell extensions was seen in both cell lines. However, as already seen by other authors in SC models for other neurological disorders, in MPS IIIA cells, those characteristics were less frequent. Regarding the expression profiles, the genes evaluated were *Nestin*, *TUBB3*, hyperphosphorylated neurofilament (*NF-H*), and neuron-specific enolase (*NSE*). In general, the increase/decrease pattern during the four weeks of neuronal induction was consistent between the controls and the disease cell lines; nonetheless, the disease cells showed consistently lower levels of all markers. This pattern was seen both in the absolute values themselves and in differences during the period of the procedure. Attention was also paid to the model’s capacity to recapitulate disease-relevant features. So, the same parameters, which were initially assessed in fibroblasts, were also analyzed after the neurodifferentiation protocol. Not surprisingly, the MPS IIIA cells exhibited higher levels of HS, a consequence of lower enzyme activity compared to the controls, further validating the disease modeling value of this kind of cells [63].

As previously stated, though, other MPSs apart from the Sanfilippo syndrome may benefit from the development of disease-specific neuronal cell models, and from the pathophysiological insights one may gain from them. Thus, some of the most striking reports on iPSC-derived neuronal and astrocytic models for MPSs actually came from MPS II. In 2019, Kobolák et al. [64] even proposed a novel neuropathology model using this approach and observed similar results regarding the impact of HS as the ones described above for MPS IIIA. They used the iPSCs originally published in a number of publications already reviewed in the previous section [54,55,56,57] that were differentiated into NPCs and terminally differentiated (TD) neuronal cells. Briefly, those iPSCs were derived from two affected siblings. As expected, both individuals shared the same nonsense mutation. Also included in this study were their mother (a carrier for the same causal mutation) and an unrelated patient with a different mutation (missense). Finally, the authors also included cells from an unrelated non-carrier, which were used as a control. At the neurodifferentiation stage, neither the patient-derived nor the healthy cells had differences in the expression of specific neuronal markers. Briefly, for NPCs the authors assessed Nestin, Sox1, and PAX6; for terminally differentiated neuronal cells, on the other hand, they checked TUBB3, MAP2, and neurofilament 200 KDa (NF200). An exhaustive characterization of those cells was conducted, showing that mature neurons exhibited postsynaptic density protein 95 (PSD95) expression, an indicator of activated synapses. Astrocytes, on the other hand, were shown to be positive for GFAP and aquaporin 4 (AQP4) markers [64]. Interestingly, according to these authors’ results, the proliferation capacity of NPCs seems to be a distinctive factor between the controls and the patients’ cells, once, after 8 passages, the proliferative capacity of the MPS II-derived cells slowed down or even stopped and the PAX6 and Sox1 expression decreased, independently of bFGF and EGF presence in the cell culture. Meanwhile, the control-derived NPCs maintained the proliferation rate up until passage 12. Actually, the authors considered this event to be related to the overall MPS II brain pathology: in normal conditions HS binds to growth factors at a proper rate, not harming their proper function. However, in the case of storage, the accumulated HS usually binds to growth factors at a higher rate, including to the one with a key role in NPC proliferation, FGF2. This link prevents the accomplishment of the transcription factor function. As a response, the cells start to differentiate into neurons, occurring in the appearance of anticipated neurites when compared with control cells [64]. 

One of the essential aims of this work was to verify if some of the disease hallmarks were already present in the NPC stage. Thus, the authors performed several analyses and, remarkably, they realized that GAG accumulation was not evident. Interestingly however, it was even reduced compared to both controls (carrier and non-carrier). They hypothesized that this phenomenon could be related to the lower levels of the early endosomal marker RAB5 (which is translated in a lower endocytosis level), as well as to the normal levels of the late endosomal marker RAB7 and the lysosomal marker cathepsin D, in addition to the higher LAMP-2 expression. The existence of those factors is reflected in functional exocytosis by patients’ cells: GAGs and GAG fragments are expelled to the extracellular space, which could explain the appearance of GAG accumulation in cerebrospinal fluid. This whole pattern changed, however, when mature neurons and astrocytes were analyzed. In fact, for those mature neurons and astrocytes differentiated from cells harboring the frameshift/PTC mutation, GAG accumulation was (quite) evident. Importantly, however, the levels of Rab7, Rab5, and LAMP-2, were still similar to those observed in controls, indicating a non-influence of the endosomal–lysosomal system on substrate accumulation. It should also be stressed that for the cells harboring the missense mutation all assessed parameters were comparable to those seen in the controls. While somehow unexpected, these results highlight the intrinsic potential of these sort of cell-based patient-derived models as they allow for more accurate comparisons between the effect of different disease-causing mutations of several subcellular parameters, ultimately allowing for more precise genotype–phenotype correlation. Also noteworthy, regardless of the analyzed genotype all terminally differentiated neuronal cells (neurons and astrocytes) showed a significant increase in the autophagy marker LC3-I, revealing the involvement of this pathway in disease cytopathology. Additionally, an accumulation of autophagosomes, as well as a lower ratio of LC3-II/LC3-I, was also detected [64].

Regardless of the cell differentiation status, a common point in the cytopathology of MPS II from NPCs and TDs was the presence of ER stress with the occurrence of dilated ER cisterns. In NPCs, the authors observed a significantly higher level of XBP1, a well-known ER stress marker. For TDs, even more events related to this stress were observed, namely depletion of ER luminal Ca^2+^ storage, higher ion concentration in the cytoplasm, and a higher sensitivity to apoptosis. Concerning cell death, they noticed a higher rate of apoptosis in astrocytes rather than other TDs. It is known that this cell type plays an important role in supporting the differentiation and survival of cortical neurons. Therefore, if they are not functional, cell death and neurodegeneration may occur [64].

Furthermore, for MPS I, a few studies exist where iPSCs were differentiated into NSCs and from which curious insights were gathered. An interesting study was performed in 2018, by Swaroop et al. [65], where after generating iPSCs and NSCs from all MPS I subtypes, the authors addressed the question of whether those three subtypes could be distinguished from each other, while extensively characterizing each one of them. In the characterization step, they observed a normal iPSC and NSC morphology, karyotype, and growth rate in all three. Still, differences among the MPS I subtypes were quite evident, when it came to the diseases’ hallmarks. Regarding enzyme activity, for example, all NSC-MPS I types exhibited a lower rate when compared to controls. However, the levels observed in the Hurler-derived cells were remarkably lower than in the others. The same happened when DS and HS accumulation and lysosomal enlargement were evaluated: the values for the Hurler subtype were much higher. Also noteworthy, when the authors compared those cell lines using differential expression (DE), about 3036 genes were found to be significantly changed between patients and controls. Remarkably, however, out of those, 42% were exclusive to the Hurler syndrome. Not surprisingly, those genes were involved in GAG homeostasis, dysregulation of the lysosomal pathway, and autophagy [65]. Overall, these results strongly support the idea that one can nicely characterize and distinguish different forms of the same disorder by evaluating iPSC-derived models, as they recapitulate the severity seen in patients at the subcellular level [65]. 

Four years later, another interesting study was conducted by Lito S. et al. [66] focusing on the most severe form of the disease alone (Hurler). In that paper, in addition to reprogramming dermal fibroblasts into iPSCs and generating NSCs, the authors went one step beyond and created an isogenic control from these iPSCs by re-establishing *IDUA* expression to avoid any type of variability that could emerge from the comparison with control iPSC lines derived from other individuals. Then, those isogenic cells were also differentiated into NSCs. As a matter of fact, these cells showed a total functional enzyme, both in the iPSCs phase as well as when differentiated into NSCs. Through comparison with isogenic cells, they could see the most evident hallmark of these disorders: GAG accumulation. Furthermore, at the end of a three-week-long neuronal differentiation protocol (where FGF2 and EGF were removed from the media), they saw a higher migration of rescued-enzyme NSCs as well as neurite outgrowth when compared to deficient iPSC-derived NSCs in vitro. In turn, proliferation capacity during three weeks of neurodifferentiation, did not change significantly between the two cell conditions. They hypothesized that due to the strong binding properties of CS and HS when accumulation occurs, these storage products bind to molecules responsible for neurite outgrowth and cell migration, preventing their binding to the proper receptors, and accomplishing the right function. Furthermore, these aspects were accompanied by an evaluation of gene expression patterns. Biological processes associated with the pathways of TGFβ and focal adhesions, PI3K-AKT signaling, Hippo signaling, the RAP1 signaling pathway, extracellular matrix interaction, and calcium signaling were altered with around 173 downregulated and 167 upregulated genes. In general, these migration defects and gene expression changes seen in patients affected by monogenic diseases are associated with a cause–effect relationship, where the genotype presents as a cause and the phenotype as an effect. However, based on these results, the authors proposed that the reverse may also occur, presenting a bidirectional pattern [66].

### 3.3. iPSC-Derived Neuronal Cells for Drug Screening/Therapy Evaluation

As we already referred to, MPS iPSC-derived neuronal cells have been generated not only to model MPS and study their pathology. Indeed, one of their crucial goals is to work as a platform to test future therapeutics. Thus, several research and development groups, some of them already mentioned in the previous sections, have been using those cells to test a number of compounds that may allegedly hold promise for the treatment of this LSD class. 

Starting, again, with the Sanfilippo syndrome, one of the studies referred before [62], besides intending the development of neuronal and astrocytic models derived from MPS IIIC iPSCs, also aimed at testing an SRT approach that had already given positive results in MPS IIIC fibroblasts [67]. That was the work of Benetó and co-workers, back in 2015, and the approach they wanted to test consisted of the use of an siRNA against one of the genes responsible for GAG biosynthesis (the *EXTL2* gene) as a genetically triggered SRT. Still, while its application in the generated neuronal and astrocyte cells revealed a great success in the reduction in mRNA levels of this gene (about 75%), when the HS levels were analyzed in neurons, no difference in substrate accumulation could be detected. Curiously, this parameter was not measured in astrocytes, and it is actually a future perspective of this the group to test it as well. A few years ago, this team also reported an siRNA-driven SRT approach against *EXTL3* (another gene involved in GAG biosynthesis) with positive results in fibroblast disease cells [67], and they proposed to assess its effect in the same neuronal and astrocytic models, but, to the best of our knowledge, no follow-up studies have been published so far. Altogether, however, the results they published so far, further highlight the need to develop suitable cell models for drug testing, by clearly demonstrating that there may be significant differences between the results obtained in vitro in fibroblasts vs. neurons using the exact same therapeutic molecule. In fact, fibroblasts are the classical human cellular model in LSDs, but there are significant metabolic differences between fibroblasts and neural cell types. Furthermore, fibroblasts are dividing cells, while neurons are not. This means that even though fibroblasts accumulate undegraded materials, storage can be underestimated due to dilution by cell division, when compared to that in non-dividing cells [62].

One year later, Huang W. et al. [68] published a comprehensive work, which went all the way from the iPSC generation and characterization to the generation of (iPSC-derived) MPS IIIB neuronal cells. While it goes far beyond the scope of this review to go through the extensive characterization analyses and pathophysiological assessments the authors performed on both types of cells, we would like to briefly highlight the therapeutic assessments they made in vitro using these models. Briefly, they examined the effects of three possible therapeutic agents: ERT with recombinant NAGLU (rhNAGLU), δ-tocopherol (DT), and hydroxypropyl-β-cyclodextrin (HPBCD). When rhNAGLU was applied to NSCs, a dose-dependent decrease in enlarged lysosomes was readily observed; the same happened when testing DT and HPBCD in addition to a dose-dependent reduction in the lipidic accumulation. In fact, those two compounds have already shown positive results in Niemann–Pick disease type C and, more recently, also in other LSDs [69,70,71]. Due to this observation, both compounds were also evaluated in MPS II iPSC-derived NSCs by Hong et al. [72]. In the case of DT, the results showed a reduction in lipid accumulation after three days, but in a dose-dependent manner; in turn, when evaluating the lysosomal accumulation, only a 7% reduction was revealed. The HPBCD results were not so encouraging, since it had virtually no effect on primary and secondary accumulation. As previously anticipated, however, when NSCs were treated with a recombinant enzyme for MPS II (rhIDS), a marked reduction in lipid accumulation was also observed. 

Curiously, the effect of rhIDS enzyme was also the target of a study presented in 2018 by Rybová et al. [73]. This study also contemplated reprogramming MPS II PBMCs into iPSCs and their subsequent differentiation into NPCs, neurons, astrocytes, and oligodendrocytes. Having properly characterized all those cells, the authors moved on to evaluate the effect of rhIDS on GAG levels. Remarkably, however, their results showed that despite achieving 10-fold higher enzyme activity levels, the treatment could not reverse the exponential growth of GAG levels, even though some decrease could be seen [73].

### 3.4. Genetically Corrected MPS-Derived iPSCs

Finally, we will also mention a few studies, which have provided in vitro proof of principle on the potential of ex vivo genetically corrected iPSCs for therapeutic purposes. 

The proof-of-concept study on the therapeutic use of iPSCs for autologous HSCT was published in 2015, by Griffin and co-workers, who attempted ex vivo gene therapy using patient iPSC-derived NSCs to reverse brain pathology in MPS VII [74]. Those authors assessed the engraftment potential of MPS VII NSCs genetically corrected with a transposon vector, by transplanting those cells into a previously reported mouse model for the disease, the so-called NOD/SCID/MPS VII model. Briefly, they intraventricularly injected genetically corrected GFP-labeled NSCs into different neonatal mouse populations, either suffering or not suffering from MPS VII. Remarkably, the authors observed similar levels of cell distribution in both pathological and non-pathological contexts, demonstrating that engraftment properties are not influenced by the disease. Importantly, transplanted cells survived and remained in the immature stage (Nestin-positive) for over 4 months. However, the proliferation rate reduced dramatically with the total disappearance of proliferation markers after 4 weeks following transplantation. It is worth mentioning, that the authors chose to work with neonatal mice for these initial assessments because they provide a more hospitable environment for engraftment relative to the adult brain. Then, to test whether similar results could be obtained in older animals, they injected ex vivo corrected MPS VII iPSC-NSCs in diseased mice’ adult brains. Again, the immature stage remained with a Nestin-positive pattern. In the adult mice, however, the authors also addressed a number of pathological aspects, in order to address the therapeutic potential of this approach. In fact, they did detect GUSB activity but only near the injection site of the hemisphere receiving corrected cells. Additionally, they also verified a high reduction in neuroinflammation after only 1 month following transplantation in that same region. Basically, they showed that xenotransplantation of ex vivo corrected MPS VII-derived NSCs into a mouse homolog of the human disease can reverse pathologic lesions surrounding the engrafted cells. However, more relevant than the particular results they saw in this disease and their accurate analysis, are the innovation potential they hold and the new avenues they open by showing that genetically corrected iPSC-derived NSCs may indeed have the potential to treat MPSs [74]. 

Then, in 2018, Clarke et al. described a somewhat similar approach, attempting to use genetically corrected NSCs derived from iPSCs as a transplantation approach to the treatment of MPS IIIB [75]. Briefly, *Naglu*^−/−^ mouse embryonic fibroblasts were reprogrammed into iPSCs and later differentiated in NSCs. Those cells were then corrected ex vivo, through lentiviral transduction of the full-length human *NAGLU* cDNA. This led to an obvious overexpression of the gene in the corrected NSCs, which resulted in a 4-fold increase in enzyme activity and in a 14-fold higher level of secreted NAGLU when compared to wild-type cells. Importantly, before they attempted HSCT of those genetically corrected cells, the authors confirmed in vitro whether secreted NAGLU could enter *Naglu*^−/−^ cells in an M6P-dependent way, and verified that corrected cells were indeed able to “cross-correct” enzyme-deficient ones. Additionally, they also addressed whether there was a difference in lysosomal enlargement between genetically corrected NSCs and unmodified *Naglu*^−/−^-derived NSCs. Curiously, they could not see any differences. However, when both cell lines were allowed to differentiate into mature neural cells, the ones derived from genetically corrected NSCs did show a significant decrease. Only then did the authors move to in vivo studies. Basically, they repeated virtually the same experiments previous teams had performed before: ex vivo genetically modified cells were injected into newborn *Naglu^−^*^/−^ mice to understand whether they would promote an amelioration of the animals’ phenotype. However, there is one remarkable aspect about this study that should be highlighted: this team evaluated two independent protocols, intracerebroventricular (ICV) and intraparenchymal (directly in the striatum), and the pathological aspects they analyzed were microglial activation, astrocytosis, and lysosomal dysfunction/storage material. These aspects were analyzed using immunostaining of CD68, GFAP, and LAMP-1, respectively [75]. 

Again, we will not review in detail all their observations, but we would like to stress that, from this team’s observations regarding the two administration routes attempted, intraparenchymal was the one shown to have better engraftment. Still, it should be stressed that, at 2 months of age, there was high variability in the pathophysiology results in both ICV and intraparenchymal approaches. Importantly, however, the follow-up results after long-term transplantation of the corrected NSCs into *Naglu*^−/−^ mice were much more conclusive. The evaluation of the long-term effect was performed after 9 months following transplantation via the intraparenchymal administration route. In general, NAGLU activity was detected in the majority of engrafted animals. Furthermore, all pathological hallmarks evaluated were more pronounced in non-transplanted *Naglu*^−/−^ mice. In grafted *Naglu*^−/−^ mice, however, CD68 and GFAP levels were significantly lower in some regions of the brain. A similar pattern was observed after LAMP-1 staining, which correlated with a significant decrease in storage material. Furthermore, transplanted mice displayed a reduction in astrocyte activation, accompanied by a complete prevention of microglial activation not only within the area of engrafted cells but also on its neighboring regions. Quite remarkably, beneficial effects were observed along the rostrocaudal axis of the brain. Altogether, this study provided evidence that the transplantation of genetically corrected iPSC-derived NSCs, may indeed represent a potential treatment for MPS IIIB, and this is particularly relevant since no approved therapeutic approach exists for this neurological MPS [75,76]. 

The latest ex vivo gene therapy experience to be performed in MPS models is extremely recent. It was published in 2022 [76] and took advantage of results for MPS IIIB we just reviewed [75]. In fact, the same team, which originally published the proof of principle on the potential of ex vivo corrected NSCs to positively impact the brain neuropathology in *Naglu*^−/−^ mice, later extended that study by using a modified Naglu enzyme with the fusion protein IFGII (named NAGLU-IGFII) for the ex vivo correction of the NSCs. This modified/chimeric enzyme, had already been described to allow a greater cellular uptake via IGFII binding sites on the M6PR. Again, the overall process of NSC generation was performed, as well as lentiviral transduction of the *NAGLU-IGFII* sequence. Having confirmed that the modified NAGLU-IGFII enzyme could also be secreted and taken up, just like the unmodified enzyme they had previously reported [75], the authors moved on to in vivo studies. Briefly, they engrafted modified cells into the brain of newborn mice and evaluated the long-term therapeutic effect of that approach, 9 months post-transplantation. First, they confirmed the remaining capability of engrafted NSCs to generate different subtypes of CNS-associated cells through positive staining of several markers: NeuN and MAP2 for neurons; GFAP for astrocytes; and O4 for oligodendrocytes. Once more, the success of the engraftment could be better since there was a high variability in the enzyme activity between sections of the brain in different animals. However, the range of enzyme activity was increased by 10%, compared to *Naglu*^−/−^ mice, which could be promising since it is reported that sometimes only an increase of 1–5% is sufficient for a proper enzyme activity correction. In the case of pathophysiological events, glial activation and storage accumulation, measured, respectively, through the staining of CD68/GFAP and LAMP-1, revealed a pattern similar to that of wild-type animals. Both effects were more pronounced closer to injection sites [76]. Furthermore, the authors also assessed a parameter which had not yet been looked at in previous studies: the downregulation of MAP2. MAP2 is now known to have a relevant/significant role in the microtubule stabilization of dendritic processes. Its downregulation is heavily associated with dementia in Alzheimer’s disease. Dementia is also a primary symptom in MPS IIIB and, remarkably, when *Naglu*^−/−^ mice were stained for MAP2, the results showed that MAP2 was reduced when compared to wild type. Nine months post-transplantation this downregulation was actually reversed, with treated animals presenting MAP2 levels similar to those observed in *Naglu*^+/−^ mice. Moreover, the accumulation of aggregates of synaptophysin, which is a known indicator of axonal damage in inflammatory conditions, was higher in *Naglu*^−/−^ mice than in wild-type and engrafted animals [76].

Overall, even though the efficacy of this therapeutic approach must be improved to reach all brain sections and counteract the Sanfilippo-associated neuroimmune response throughout the whole brain, truth is that, once more, this team has gathered evidence on the possibility of ex vivo gene therapy, with remarkable ameliorated MPS IIIB phenotypic aspects. Moreover, this was the first report documenting a significant reduction in the neuronal marker MAP2 and accumulation of synaptophysin-positive aggregates, both well-known to be related to neuropathophysiology [76].

Then again, even MPSs, which already benefit from the existent ERTs, may ultimately benefit from this sort of approach. Therefore, ex vivo gene therapy experiments have also been performed in MPS I. In fact, in 2019, Miki et al. [77] generated iPSCs from *Idua^−/−^* mouse embryonic fibroblasts. Then, the authors performed the ex vivo correction of those cells using CRISPR/Cas9 technology and verified that the resulting levels of enzyme activity were significantly restored with values comparable to the wild-type iPSCs. While exploratory and not yet attempted in vivo, these results further validate the overall potential of iPSCs and iPSC-derived cells for gene therapy in MPSs. 

Table 1 summarizes works performed in MPSs using iPSC technology.

## 4. A Hypothetical Alternative Approach to Model Mucopolysaccharidoses

**Figure 2:**
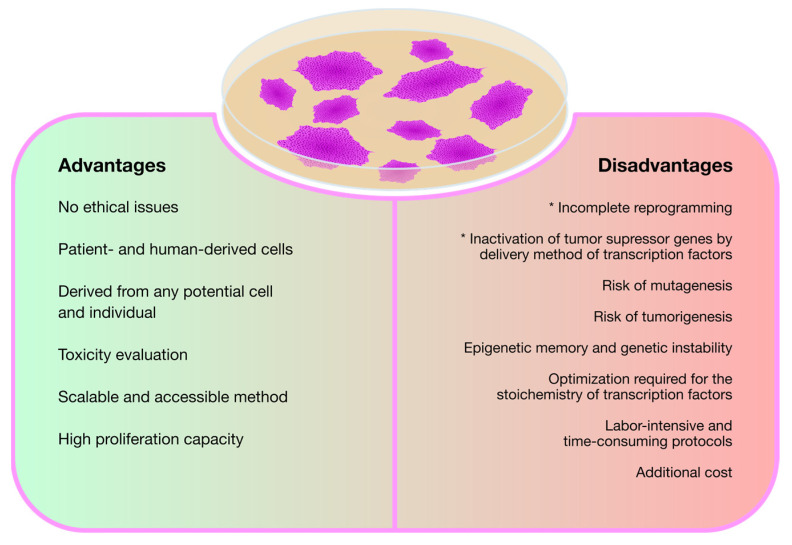
Advantages and limitations of iPSCs. Even though extensively reported in the literature, the issues marked with (*) have been largely solved thanks to the most recent technical improvements of the iPSC generation protocols.

Regardless of their ultimate purpose, the rationale followed in virtually all the studies reviewed so far is the same: first, differentiated cells from patients with the target disease are reprogrammed into iPSCs and, then, differentiated again but into disease-relevant cell lines, thus creating a viable cell model for neuronopathic MPS. This technology, as described above, is undoubtedly contributing to increase the knowledge on the pathophysiology of MPSs with neurological involvement and, consequently, with no treatment available. Nevertheless, while iPSC technology proves to be quite valuable and promising, it also involves some disadvantages. Those positive and negative considerations are recapitulated in Figure 2.

**Figure 3:**
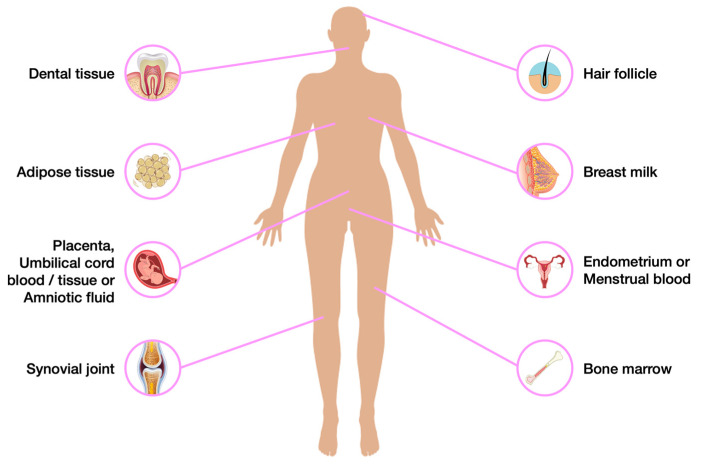
Different sources of mesenchymal stem cells (MSCs).

That is why alternative protocols and additional sources of SCs should also be considered, especially those which are naturally-occurring (Figure 3).

**Figure 4:**
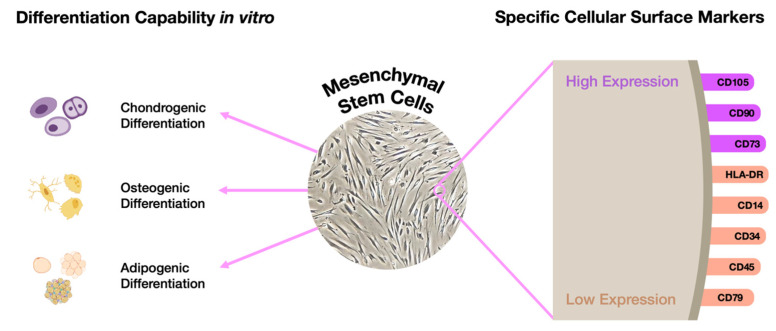
Minimal requirements for identification of mesenchymal stem cells (MSCs).

An excellent option would be to take advantage of patients’ MSCs, reducing the possibility of errors and avoiding the long, laborious, and expensive pluripotency induction phase. In fact, those cells represent a suitable alternative since they can be differentiated into any of the three germ layers: endodermal, mesodermal, and ectodermal, as long as they are cultured in proper media. To be considered an MSC, the cell needs to fulfill a number of criteria (Figure 4).

Bone marrow mesenchymal stem cells (BMMSCs) are the most often used ones. However, the patients’ wellness remains an essential issue, due to invasive procedures [79,80].

An interesting study [81] in 2000 introduced a possible new source of SC to the world: the dental pulp. The dental pulp is an oral non-mineralized tissue with various cell types, localized in the central pulp cavity, and mostly comprises soft tissue with nervous/vascular lymphatic elements [82]. Inside it, we may find the so-called dental pulp stem cells (DPSCs). Those cells have an ectodermal origin derived from neural crest cells [83], more specifically from peripheral nerve-associated glia [84]. In that original study [81], those recently discovered SCs were compared to BMMSCs, and the evidence the authors gathered showed that those DPSCs exhibit a higher proliferation rate when compared to BMMSCs, while expressing the same pluripotency markers. Thus, this pivotal study became a launching pad for the subsequent exploration of these cells. The impossibility of generating adipocytes in the original study was the only lack in classifying DPSCs as MSCs. However, over the following years, more evidence was gathered proving their stemness. Ultimately, in 2002, the same group that originally assessed their MSC features, was actually able to promote the adipogenic differentiation of those cells, using a more specific induction medium. They also confirmed that human DPSCs are capable of self- renewal after an in vivo transplant [85].

**Figure 5:**
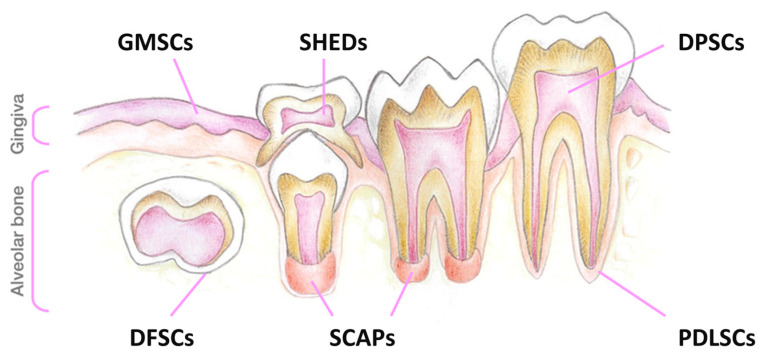
Schematic drawing illustrating the sources of dental mesenchymal stem cells (DMSCs) in the oral cavity. Abbreviations: GMSCs, gingiva-derived MSCs; DFPCs, dental follicle progenitor cells; SHED, stem cells from exfoliated deciduous teeth; SCAPs, stem cells from the apical papilla; DPSCs, dental pulp stem cells; PDLSCs, periodontal ligament stem cells.

After a few years of constant research, a terminology was established that is still used today, which allows us to distinguish between the different SC populations that reside inside the dental tissue (Figure 5). Indeed, depending on the source of the oral cavity from which they are extracted, five different types of SCs may be distinguished: DPSCs, stem cells from deciduous teeth (SHEDs) [86], stem cells from apical papilla (SCAPs) [87], periodontal ligament stem cells (PDLSCs) [87], and dental follicle stem cells (DFSCs—precursor cells of PDLSCs) [87,88,89]. Collectively, for their confirmed MSC properties, these cells may be denominated as dental mesenchymal stem cells (DMSCs).

In addition to the different oral cavity source, we can distinguish those SCs by their proliferation rate and potential to differentiate into the several cells. Regarding the proliferation rate, the follicle-derived ones seem to have the highest, closely followed by SHEDs, SCAPs, PSLSCs, and DPSCs [90,91,92,93,94,95,96,97]. In Table 2, we review some experiments performed so far, to identify the best cell type for each kind of differentiation.

We are now in 2023, and the existence of these different SC populations has now been known for over 20 years. So, DPSC and SHEDs in particular, have been generated from pulp for some time. Nevertheless, the majority of the studies involving those cells have focused on their differentiation into chondrocytes for dental repair, with the eventual goal of re-growing teeth from multipotent DMSC [85,98]. Also addressed by a few teams is the potential they hold for stroke therapy. The first study to investigate DPSCs in an animal model of stroke dates back to 2009 and used a mechanical extraction method to obtain cells from human third molars. The cells extracted from those teeth were shown to efficiently express the nuclear receptor related 1 protein, which is essential for the dopaminergic system of the brain, and to promote, when transplanted, functional motor recovery [99]. After this pivotal study was performed, a few others followed, always relying on the use of SCs from different dental pulp sources, and being tested in vivo in rat models of focal cerebral ischemia via the transient occlusion of the middle cerebral artery. While it falls completely out of the scope of this review to summarize all those studies, it is worth mentioning that most of them showed really promising results (reviewed in [100]). Curiously, those cells have been shown to enhance post-stroke functional recovery through a non-neural replacement mechanism, i.e., via DPSC-dependent paracrine effects ([101]; reviewed in [100]). This is probably one of the reasons why these sorts of cells have been addressed for their therapeutic potential in many other disorders, affecting various different organs such as kidney (acute renal injury [102,103] and nephritis [104]); lungs (acute lung injury [105]); brain (Parkinson’s disease [106,107], Alzheimer’s disease [108], cerebral ischemia [109,110]); spinal cord (spinal cord injury [111,112,113,114]); liver (liver fibrosis [115,116,117]); heart (acute myocardial infarction [118,119]); muscle (muscular dystrophy [120,121,122]); bone (calvarial defect [104,123,124,125], and osteoporosis [126]); skin (wound injury [127,128]); pancreas (diabetes [129,130]); eye (glaucoma [131], cornea trauma [132]); and immune system (rheumatoid arthritis [133], autoimmune encephalomyelitis [134], and systemic lupus erythematosus (reviewed in [135,136]).

**Table 2:** Published results on the multilineage differentiation potential of several DMSC populations.

Type of Differentiation	Dental Stem Cell Population	Confirmation	References
Osteogenic	DPSC, PDLSC, SHED, SCAP, DFSC	Alizarin red staining	[91,97,137,138,139,140]
	DPSC, PDLSC, SHED, DFSC, SCAP	ALP activity	[91,137,140]
	DPSC, PDLSC, SHED, DFSC, SCAP	qRT-PCR (*RunX2*, *ALP*, *COL-1*, *OSX*, *OCN*, *OPN*, *SPP1*, *BMP2*)	[91,137,138]
	DPSC, DFSC, SCAP	RT-PCR (*RunX2*, *ON*, *OCN*)	[139]
	DPSC, PDLSC, DFSC	Western blot (RunX2, ALP, OSX, OPN)	[91]
Chondrogenic	DPSC, PDLSC, DFSC, SCAP	Alcian Blue	[91,139]
	DPSC, PDLSC, DFSC	qRT-PCR (*SOX2*, *COL-2*, *ACAN*)	[91]
	DPSC, DFSC, SCAP	RT-PCR (*ACAN*, *COL1α-1*, *COL1α-2*)	[139]
Adipogenic	DPSC, PDLSC, DFSC, SCAP	Oil Red O staining	[91,138,139,140]
	DPSC, PDLSC, DFSC, SCAP	qRT-PCR (*APN*, *C/EBPα*, *FABP4*, *PPARG*, *LPL*)	[91,138]
	DPSC, DFSC, SCAP	RT-PCR (*ap2*, *PPARG*)	[139]
Neurogenic	DPSC, PDLSC	Giemsa staining	[140]
	DPSC, SHED, DFSC, SCAP	Immunostaining (MAP2, TUBB3, NF-M, NG-F, Thy1)	[138,140]
	DPSC, SHED, DFSC, SCAP	qRT-PCR (*MAP2*, *TUBB3*,*NF-M*, *NF-H*, *GFAP*, *Nestin*	[138,141]
	DPSC, PDLSC	RT-PCR (*DCX*, *NCAM*, *NCAD*, *NROUND1*)	[140]

Osteogenic markers: OPN—osteopontin; OCN—osteocalcin; OSX—osterix; ALP—alkaline phosphatase; COL1A1—collagen 1A1; SPP1—secreted phosphoprotein 1. Chondrogenic markers: COL-2—collagen 2; ACAN—aggrecan. Adipogenic markers: APN—adiponectin; C/EBPα-CCAAT enhancer binding protein alpha; FABP4—fatty acid-binding protein-4; PPARG—peroxisome proliferator-activated receptor gamma; LPL—lipoprotein lipase; ap2—adipocyte protein 2. Neurogenic markers: MAP2—Microtubule-associated protein 2; TUBB3—tubulin beta 3; NF-M—Neurofilament medium-chain; NG-F—nerve growth factor; NF-H—neurofilament heavy chain; GFAP—glial fibrillary acidic protein; DCX—doublecortin; NCAM—neural cell adhesion molecule; NCAD—neural cadherin; NROUND1—neurogenic differentiation 1.

Additionally, if it is true that, for most of these injuries, the evidence gathered so far comes from in vivo studies alone, when it comes to the use of DMSCs in oral diseases, the scenario is significantly different, with two clinical studies on pulp regeneration having been launched within the past several years that have achieved breakthroughs in humans (reviewed in [135]). Overall, the results are so good and the possibilities so vast that soon a commercial interest was found in this type of cells. In fact, due to their easy accessibility and favorable therapeutic applications, cell/tissue banking in the dental field is now a reality in several countries, with some of the most well-known ones being BioEDEN (Austin, TX, USA), Store-a-Tooth (Lexington, KY, USA), Cell Technology (Japan), or the Tooth Bank (Brownsburg, IN, USA) (reviewed in [96,142]). Additionally, as exciting as these results and perspectives may sound per se, we believe that the overall potential of these SCs goes far beyond their properties for tissue repair and regeneration. We think, as other authors have also highlighted before, these cells also hold an exceptional potential for neurogenetic disease cell modeling and basic research. In fact, the neural crest origin of DMSCs makes them an excellent proxy to study NSCs in a lab setting, and a useful source of primary cells for modeling virtually any neurological disorder at the molecular level [143]. Therefore, it will be of utmost value to better characterize different populations of patient-derived DMSCs to understand to what extent these cells will mimic the subcellular phenotype seen in iPSC-derived NSCs, for example, or even in other, more elaborate cell models, such as engineered neuronal and glial cells. Given our interest in LSDs, their monogenic nature, and the extremely high prevalence of severe neurological phenotypes in this group of disorders, we considered DMSCs an excellent model to study these disorders. Additionally, over the past few years, several successful neurodifferentiation protocols have already been described (reviewed in [144]) and the generated cells characterized by several different teams (Table 2). Still, there is clearly room for improvement, both in terms of protocol standardization and of the subsequent characterization of differentiated cell lines. Indeed, there are many differences between the different protocols, some subtle and others more obvious [145,146]. For example, besides the adherent growth of cells, also non-adherent conditions can be applied for the formation of neurospheres that will further differentiate into neuron-like cells [147,148]. There are even some published comparisons between different neurodifferentiation conditions, but more consistent data and a higher number of studies remain mandatory for more robust conclusions to be drawn. Only then will the modeling potential of DMSCs and their derived counterparts be fully unveiled.

Interestingly, while their modeling potential has never been addressed for LSDs, as advantageous as it may sound, truth is that DPSCs are not totally unknown in the field. In fact, back in 2015, Jackson et al. [149] suggested that human MSCs derived from bone marrow and dental pulp could work as an alternative to the use of hematopoietic stem cells in standard transplantation approaches for the treatment of MPSs. Similarly to what has been discussed in the last section, in which we summarized the studies published so far on MPSs using iPSCs, in this particular publication, it was the therapeutic potential of the MSCs per se, which was analyzed. Actually, none of the MSCs analyzed were derived from MPS patients. Instead, all studies were performed on MSCs obtained from healthy donors. This meant that neither the BMMSCs nor the DPSCs they established had any MPS-related enzymatic defect. Instead, all analyzed cell lines (MSCs and HSCs) were able to produce the different MPS-associated enzymes in the cell layer and secrete low levels of each and every one of them into the surrounding media, the same being true for the used HSCs. However, MSCs were found to produce significantly higher levels of the majority of MPS enzymes assayed when compared to HSCs, a result that can be considered particularly relevant for therapeutic purposes.

However, these authors achieved more than just characterizing the normal levels of MPS-related enzymes secreted by different types of wild type SCs, namely BMMSCs, DPSCs, and HSCs. They also attempted to overexpress, through lentiviral transduction, four different lysosomal enzymes in those same cell lines, to check whether their secretion levels were somewhat similar. Importantly, the evidence they gathered further supported the idea that MSCs had higher secretion and production levels of MPS enzymes when compared to HSCs. Also noteworthy, the lentiviral transduction was more efficient in MSCs compared to HSCs.

Then, the authors moved on to investigate, in vitro, the cross-correction potential of MPS enzymes secreted from those two different sorts of MSCs in MPS patient-derived fibroblasts, and, after confirming the reduction in GAG accumulation, they also verified that this cross-correction was reached in an M6P-dependent way.

Finally, they also addressed the differentiation ability of the MSCs tested, verifying that both transduced and non-transduced cells maintained that capacity, with only slight differences in the neurogenic process, which appeared to have a slower differentiation pattern in transduced MSCs. As expected, however, MSCs derived from dental pulp had a premature upregulation of mature neuronal markers, when compared to those derived from bone marrow.

Altogether, these results provided the in vitro proof of principle of the therapeutic potential of DPSCs and BMSCs as an isolated therapy, or even a combined therapy with the standard HSCTs. To the best of our knowledge, no follow-up studies or in vivo assessments have yet been published on this subject, even though its overall results seem extremely promising.

**Figure 6:**
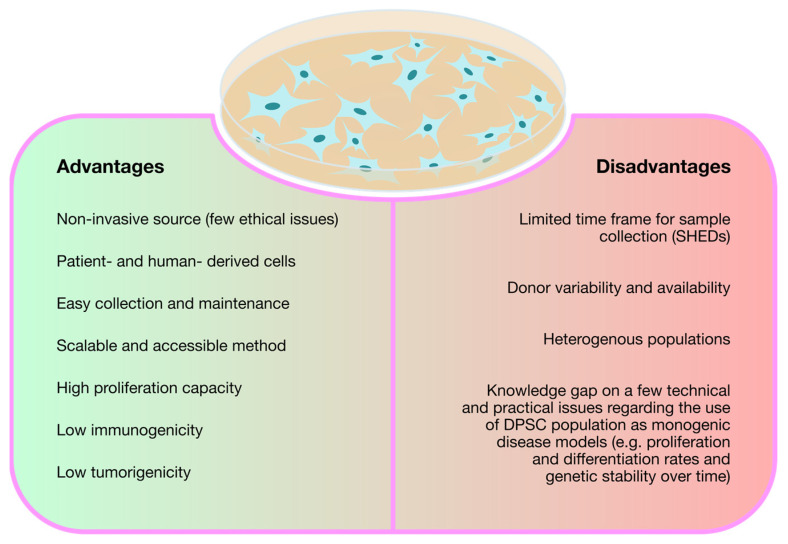
Advantages and limitations of dental pulp stem cells (DPSCs).

To the best of our knowledge, MPS patient-derived DPSCs have never been used for differentiation into specific cell types even though they represent a natural source of SCs that may be used to investigate human diseases, especially the infantile forms of these disorders (Figure 6). In fact, taking into account that the most severe forms of MPSs are pediatric, there is one particular population of SCs in the dental pulp that seems particularly suitable to study them: SHEDs. Among their numerous advantages, which include a high proliferation rate and the greater tendency to generate both skeletal and brain cells, SHED collection does not require the active removal of teeth, only their natural fall, and this is certainly an advantage for children who may already be dealing with undue stress and pain.

It is also worth mentioning, that while this review focuses on the insights one can get on the neuropathology of MPSs by studying iPSCs, and we have only commented on the neuronal differentiation potential of SCs from different sources, such as the DPSC, their differentiation capacity to osteogenic fates is also known and successful protocols have been published. This is quite relevant for MPS disease modeling because some of these diseases present with a marked skeletal phenotype, which fails to be corrected by the currently available therapies. Thus, by implementing the method envisaged here, one may also pave the way for additional applications of DPSCs. For example, we may easily foresee their differentiation into chondrocytes, one of the major components of cartilage and primary site of accumulation in several LSDs.

In general, the higher the number of genotypes we collect, the larger the spectrum of future applications our DPSC-derived LSD neuronal cultures may have not only in our lab but also for other researchers in the field. In addition, with the advances of new gene editing technologies, such as CRISPR/Cas, base editing, prime editing, and the "older" transcription activator-like effector nucleases (TALEN) and zinc finger nucleases (ZFN), the possibility to generate pairs of isogenic lines that facilitate the study of the function of a given gene and the role that different mutations play in the pathophysiological mechanisms of the respective diseases arose. This approach has been increasingly applied to iPSC lines and could also be very useful in the case of our DPSC-derived cell lines. However, the value of these cutting-edge technologies goes far beyond the generation of isogenic cell lines to account for individual variability and allow more precise conclusions regarding gene function or mutation-specific effects in one particular phenotype. They bring along invaluable possibilities, and greatly expand the catalogue of methods which may be used not only for basic but also for translational research. In the specific case of the CRISPR/Cas technology, for example, it has become a relevant platform due to its simple configuration, using advanced algorithms and web-based tools, that allow to analyze its efficiency in recognizing the sequence of interest as well as possible off-targets. Therefore, many advances have been made not only in disease modeling but also in its potential application to gene therapy, namely in combination with stem cells [150]. Recently, Ahumada-Ayala et al. [151] reviewed a number of studies on therapeutic approaches using CRISPR/Cas, namely in hematological, hemato-oncological, cardiovascular, neurological, infectious, and musculoskeletal diseases. Some of these studies show the importance of CRISPR as a promising therapeutic approach, but also show the challenges that still need to be overcome, such as safety concerns arising from toxicity due to the double-strand breaks, chromosome loss, and genomic instability, the potential for off-targets, as well as bioethical implications. Nevertheless, given the rapid advances in these cutting-edge technologies, there is hope that gene editing platforms such as CRISPR/Cas can be used for correction of genetic mutations and restoration of functional deficiencies, offering a therapeutic alternative for life-threatening diseases.

Still another naturally occurring source of SCs are human urine-derived stem cells (USCs), a type of MSCs with proliferation and multi-potent differentiation potential that can be readily obtained from voided urine using a non-invasive protocol and with minimal ethical restriction. These cells express surface markers of MSCs, but not of hematopoietic stem cells, express the stemness-related genes *Nanog* and *Oct3/4*, and show telomerase activity, not forming teratomas in vivo after being subcutaneously implanted in nude mice [152,153,154,155]. When cultured in appropriate media, USCs may differentiate into endothelial, osteogenic, chondrogenic, adipogenic, skeletal myogenic, and neurogenic lineages. Interestingly, USCs may be established from individuals of any age, despite Gao et al. having shown that those isolated from children (5 to 14 years old) show higher proliferation, lower tendency to senescence, and stronger osteogenic capacity than those from middle-aged (30 to 40 years old) and elderly (65 to 75 years old) individuals [154]. This property allows to significantly expand the cohort of patients accessible to be studied. Overall, USCs are yet another alternative source of SCs that can be used as a valuable in vitro model to study genetic diseases, with potential applications in regenerative medicine, cell therapy, diagnostic testing, and drug screening [156].

Finally, it is important to stress that even though in this review we have only focused attention on cell models, the unquestionable role that animal models continue to play in disease modeling cannot be forgotten. Indeed, despite the cellular models here described displaying numerous advantages for both basic and translational research, namely the fact that they cover the genetic diversity needed to fully model human genetic diseases, in vivo models are fundamental not only to explore and interpret the pathophysiology of the disease in the context of the whole organism, but also the cognitive and behavioral aspects. This is particularly important in diseases with marked/severe neuronal involvement such as MPSs. In fact, only animal models allow for a comprehensive analysis of the CNS as a whole, starting with the molecular and cellular examinations all the way up to the macroscopic anatomical and behavioral observations. As reviewed by Megagiannis et al. [157], it is necessary to go through the animal model phase in order to evaluate if a new therapeutic approach is safe and effective at all levels, including in the behavior and in social interaction. In particular, the latest evaluation is impossible to obtain using in vitro models. However, even the type of animal model that will be used for a certain disease must be taken into account, as its pathophysiological pathways may differ from those in humans, especially in the species further down the phylogenetic tree. For example, symptoms related to the complexity of cognitive function and behaviors in humans are difficult to model in rodent species, and therefore their interpretation must be handled with great care and caution [157]. Differences between species may impede the therapeutic translation to humans. In this regard, the use of higher order animal models can be invaluable, and gene editing can also play a key role in this case by generating specific mutants. Choosing the models for their genetic and evolutionary relevancy to humans represents an important step in adequate disease modeling. Therefore, it is the combination of animal models together with relevant cellular models derived from human patients that offer a closer look at human cells that may ultimately serve as a platform both for unraveling the pathophysiological mechanisms underlying the disease and for translating new therapeutic approaches into preclinical testing.

## 5. Conclusions

Disease models are essential tools to both identify and study the pathological mechanisms that underlie the development of a disease. They are also a pre-requisite for proper drug development. Indeed, it is essential to have a relevant study model, which reproduces the pathological features of the disease to design and evaluate new therapeutic strategies. This need goes all the way, from the early in vitro assessments to the investigative in vivo pre-clinical studies.

Over the last few decades, amazing advances have been made in the attempt to model the neuropathology of MPSs in vitro, mostly relying on the establishment and subsequent differentiation of disease-specific human iPSCs. This is certainly true for the larger LSD field, where multiple studies have identified neural progenitor cell-migration and differentiation defects, substrate accumulation, axon-growth and myelination defects, impaired calcium homeostasis, and altered electrophysiological properties, all using patient-derived iPSCs (reviewed in [158]). So, not even 20 years after iPSC generation was first described and attempted, their potential to provide mechanistic insights to unravel the pathophysiology associated with neurodevelopment in these rare pathologies is well-established. However, several challenges do remain. That is why we consider it may be useful to contemplate additional sources of patient-derived pluripotent or multipotent cell lines, namely those which are naturally occurring, such as the dental pulp SCs derived from human permanent and deciduous teeth. Those cells may even allow for subsequent differentiation into mixed neuronal and glial cultures, which may be analyzed with virtually the exact same methods many authors perform to address neuropathology in MPS-derived iPSCs. Finally, regardless of the original source of the SCs we are considering, in an era where personalized medicine and mutation-specific therapeutic approaches are gaining momentum, those SC-derived models will also constitute optimal platforms for in vitro drug testing.

## Figures and Tables

**Table 1:** A summary of the works performed in Mucopolysaccharidoses (MPSs) to date using induced pluripotent stem cell (iPSC) technology.

Disorder	Affected Gene	Defective Enzyme	Stored Substrate	Subtype	Generation of MPS-Derived iPSCs				Drug Screening	Ex Vivo Gene Therapy
					Source	iPSC	NPC	Mature Neurons		
MPS I	*IDUA*	α-L-iduronidase	DS and HS	Hurler	Fibroblasts	[58,65,66]	[65,66]			
					Mouse embryonic fibroblasts	[77]				[77]
				Hurler/Scheie	Fibroblasts	[65]	[65]			
				Scheie	Fibroblasts	[59]				
					PBMCs	[65]	[65]			
MPS II	*IDS*	Iduronate-2-sulfatase	DS and HS		Fibroblasts	[63,72]	[63,72]		[72]	
					PBMCs	[54,55,56,73,78]	[64,73]	[64,73]	[73]	
MPS III	*SGSH*	Sulfamidase	HS	A	Fibroblasts	[51]				
	*NAGLU*	α-N-acetylglucosaminidase		B	Fibroblasts	[45,50,60,68]	[45,60,68]	[68]	[68]	
					Mouse embryonic fibroblasts	[75,76]	[75,76]			[75,76]
	*HGSNAT*	N-acetyltransferase		C	Fibroblasts	[53,61]	[61,62]	[61,62]	[61,62]	
MPS VII	*GUSB*	β-Glucuronidase	DS, HS, and CS		Mouse embryonic fibroblasts	[74]	[74]			[74]
